# The Effect of Diet on Cardiovascular Disease, Heart Disease, and Blood Vessels

**DOI:** 10.3390/nu14020246

**Published:** 2022-01-07

**Authors:** Hayato Tada, Masayuki Takamura, Masa-aki Kawashiri

**Affiliations:** Department of Cardiovascular Medicine, Graduate School of Medical Sciences, Kanazawa University, Kanazawa 920-1192, Japan; masayuki.takamura@gmail.com (M.T.); mk@med.kanazawa-u.ac.jp (M.-a.K.)

**The Effect of Diet on Cardiovascular Disease, Heart Disease, and Blood Vessels**

Cardiovascular disease (CVD), including coronary artery disease, heart disease, arrhythmias, and other types of vascular diseases, are one of the leading causes of death across the world [1]. It is estimated that approximately half of the variabilities of CVD appear to be attributed to genetics [2,3]. In other words, the other half of them have been attributed to acquired factors, including diet. It is of note that even a genetic predisposition to CVD can be canceled out by a healthy lifestyle [4]. In this regard, it is important to acknowledge that acquired factors, including diet, are causally associated with CVD. Based on these facts, important papers are presented in this Special Issue entitled “The Effect of Diet on Cardiovascular Disease, Heart Disease, and Blood Vessels”.

**Omega-3 Polyunsaturated Fatty Acids (n-3 PUFA) and CVD**

It has been suggested that our diet has a great impact on our physical function and body metabolism. Among numerous nutrients, a lot of attention has been paid to omega-3 polyunsaturated fatty acids (n-3 PUFA) that can be found in fish oil. They play important roles in various cellular functions, including signaling, cell membrane fluidity, and structural maintenance. They also regulate inflammatory processes that lead to the development of CVD. Epidemiological studies have suggested that the intake of n-3 PUFA appears to have cardioprotective effects [5,6]. Furthermore, several randomized controlled trials have suggested that supplementation on top of statins can further reduce cardiovascular risk [7,8]. The beneficial effect of n-3 PUFA has been attributed to the lowering of serum triglyceride levels; however, there appear to be other “pleiotropic” effects beyond triglycerides. Gonçalinho et al. identified one of the potential cardioprotective properties of n-3 PUFA [9]. They investigated the association between n-3 PUFA within erythrocyte membranes and established cardiovascular risk factors and found that n-3 PUFA in erythrocyte membranes are independent predictors of cardiovascular risk, comprised of multiple elements that are associated with CVD. This study suggests that n-3 PUFA contributes not only to the reduction of serum triglyceride levels but also to the modification of classical cardiovascular risk factors, such as hypertension and hyperglycemia. On the other hand, Jiang et al. nicely summarized a meta-analysis of prospective cohort studies that investigated if fish and n-3 PUFA intake are associated with reduced CVD risk [10]. It is important to note that they performed independent meta-analyses on fish intake and n-3 PUFA intake and found that both were significantly associated with reduced CVD risk. Finally, they concluded that 20 g of fish intake or 80 mg of n-3 PUFA intake per day was associated with a 4% reduction in CVD-related mortality. This study clearly suggests that the cardioprotective effect of fish intake appears to be mostly attributed to n-3 PUFA. In addition, their dose-dependent association supports the notion that the amount of intake and their serum levels are important contributors to the cardioprotective effects of n-3 PUFA supplementation. Accordingly, it may be reasonable to think about the baseline dietary pattern and serum n-3 PUFA levels of patients when considering endorsing the intake of fish or n-3 PUFA and the quantity to be taken. 

On the other hand, the intake of trans fatty acids (TFA) has been associated with dyslipidemia, type 2 diabetes, CVD, and all-cause mortality [11]. As such, dietary guidelines are now recommending the non-consumption of TFAs. There are studies suggesting that TFAs are associated with dyslipidemia, type 2 diabetes, and other cardiometabolic disorders; however, Iino et al. carried out a unique study focusing on the HDL cholesterol uptake capacity. Despite the fact that statins (which can reduce LDL cholesterol) are associated with reduced CVD risk, we are still facing the reality of the so-called “residual risk” of statins [12]. There are a number of biomarkers that have been identified as such residual risk factors, including triglycerides, lipoprotein (a) (Lp(a)), and inflammation [13,14,15]. However, recent studies have suggested that the function of HDL, rather than HDL cholesterol, appears to be one of the most important residual risks for CVD [16]. Among the many functions of HDL, reverse cholesterol transport, also known as HDL cholesterol uptake, is the most important function in the field of preventive cardiology. In this Special Issue, they used s unique strategy for the measurement of HDL cholesterol uptake capacity in humans and found that elaidic acid, which is one of the TFAs, was associated with the inhibition of HDL cholesterol uptake and the maturation of HDL. This is strong evidence of the fact that fatty acids are involved in an important process of the development of atherosclerosis; therefore, it should be quite reasonable to accept it as a biomarker or even a source of cardioprotection.

**Salt Intake and CVD**

There is no doubt that hypertension is one of the leading causes of CVD. There is much evidence to support this assertion, including epidemiological studies, animal models, and randomized controlled trials [1]. Among several important factors that contribute to hypertension, the intake of salt is evidently an important one. We know that a higher intake of salt is associated with a higher risk of hypertension, and reducing one’s salt intake can protect against the development of hypertension. However, there are also several important sensitivity factors associated with salt intake and the development of hypertension, including genetic factors and acquired factors, such as dietary habits other than salt intake. In this Special Issue, Levanovich et al. performed an interesting experiment using rats, showing that the consumption of 20% fructose during adolescence predisposes to salt-sensitive hypertension [17]. Importantly, they also suggested that dietary fructose intake plus a high-salt diet during this early phase leads to vascular stiffening and left ventricular diastolic dysfunction, which are both highly associated with heart failure. The underlying mechanisms are still unclear; however, it is now clear that our diet affects hypertension as well as the risk of heart failure.

**Gut Microbiota and CVD**

Recent studies have suggested that the gut microbiota is associated with a variety of diseases, including CVD. Although they are also affected by some genetic factors, the main factor contributing to our microbiota should be our diet. In this Special Issue, Bin-Jumah et al. nicely summarized recent findings on this matter [18]. Investigations have indicated that the gut microbiota is involved in the pathogenesis of CVD and can be considered as one of its causative factors. The gut microbiota appears to have multiple functions in humans, including energy production, maintaining intestinal homeostasis, enhancing the absorption of drugs, immune responses, defense from pathogens, and the production of microbial products, such as vitamin K, nitric oxide, trimethylamine-N-oxide (TMAO), and lipopolysaccharides. Among these properties, Bin-Jumah et al. summarized the association between TMAO and heart failure and showed that TMAO, a metabolite of the gut microbiota, may have interesting perspectives regarding how this particular metabolite contributes to the development of heart failure. They also suggested that the excessive intake of the choline of L-carnitine, which contains an intermediate precursor (TMA) of TMAO, may be harmful, especially among elderly people who have dysbiosis and muscle disorders.

**Obesity and CVD**

We know very well that obesity, which is greatly affected by our dietary habits, is also a major risk factor for CVD [1]. However, there is a huge gap between Asians and Caucasians in terms of the definition of “obesity”. In addition, there is a paucity of data on this subject in the Asian population, where the average body mass index is much lower than that of the Caucasian population. In this Special Issue, Shiozawa et al. conducted analyses investigating an association between body mass index and stroke in the Japanese population using large health insurance databases comprising more than two million individuals. They found that overweight and obesity were associated with a greater risk of stroke and ischemic stroke in both men and women [19]. They also found that underweight, overweight, and obesity were associated with a higher risk of hemorrhagic stroke only in men. Thus, it seems that there are some gender gaps in terms of the effects of weight on CVD risk.

**Lifestyle Risk Score and CVD**

Finally, there is a growing trend to comprise the “risk score” in risk assessments for any conditions, such as polygenic risk scores comprising a number of common genetic variations [20]. Given that any type of CVD is associated with multiple factors, it is reasonable that such scores perform better than any single variable or parameter. Currently, the American Heart Association is advocating for the Life’s Simple 7 (LS7), which consists of 7 modifiable lifestyle behaviors and medical factors, including diet, obesity, physical activity, smoking status, blood pressure, cholesterol, and glucose level) in order to reduce the prevalence of CVD and stroke [21]. This score is quite useful because it consists of simple variables that can be obtained anywhere in the world; therefore, it can be applicable to people of all ethnicities. In this Special Issue, Nishikawa et al. investigated the association between Life’s Simple 7 scores among Japanese citizens and the incidence of atrial fibrillation (AF). They found that healthy lifestyle scores were associated with lower incidence rates of AF [22]. Interestingly, this trend is more remarkable among younger generations than among older generations, clearly suggesting that interventions for lifestyle factors may be better recommended for younger individuals in whom we can expect more benefits. 
